# Reaction of endogenous Coenzyme Q_10_ with nitrogen monoxide and its metabolite nitrogen dioxide

**DOI:** 10.1080/13510002.2019.1647005

**Published:** 2019-07-26

**Authors:** Paola Astolfi, Jean-Louis Clément, Didier Gigmes, Tatiana Armeni, Patricia Carloni, Lucedio Greci

**Affiliations:** a Dipartimento SIMAU, Università Politecnica delle Marche, Ancona, Italy; b Institut de Chimie Radicalaire, UMR 272, Aix Marseille Université, Marseille, France; c Dipartimento DISCO, Università Politecnica delle Marche, Ancona, Italy; d Dipartimento D3A, Università Politecnica delle Marche, Ancona, Italy; e Dipartimento DISVA, Università Politecnica delle Marche, Ancona, Italy

**Keywords:** Coenzyme Q_10_, nitrogen dioxide, nitroxide, isoprenic chain, addition reaction, mitochondria, liposomes, plasma

## Abstract

**Objectives:** Coenzyme Q_10_, incorporated in DOPC lyposomes or naturally present in liver bovine mitochondria or in human blood plasma, was reacted with nitrogen dioxide ^•^NO_2_ or with a ^•^NO/^•^NO_2_ mixture.

**Methods and Results:** The reaction course was monitored by Electron Paramagnetic Resonance (EPR) spectroscopy and in all cases the formation of a di-*tert*-alkyl nitroxide was observed, deriving from the addition of ^•^NO_2_ to one of the double bonds, most likely the terminal one, of the isoprenic chain. The rate constant for nitroxide formation was also determined by EPR spectroscopy and an initial rate of ca. 7 × 10^−8 ^M s^−1^ was obtained.

## Introduction

**Scheme 1.**
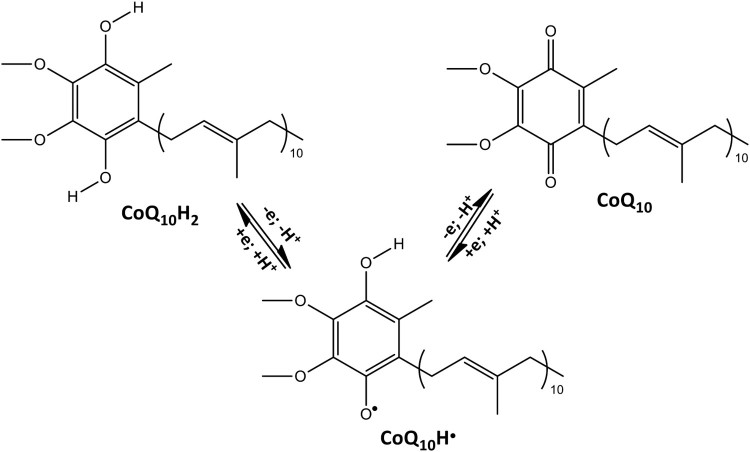
Interconversion between CoQ_10_H_2_ and CoQ_10_.

Coenzyme Q_10_ (CoQ_10_) is a component of the electron transport chain present in most eukaryotic cells and primarily in the mitochondria. It participates to the aerobic cellular respiration, generating ATP [1,2]. In the interconversion from the reduced CoQ_10_H_2_ to the oxidized CoQ_10_ (Scheme 1) electrons are transferred to the enzyme complexes of the chain responsible for the oxidative phosphorylation and ATP production. It follows that organs with the highest energy requirements, such as heart, liver and kidney, have the highest CoQ_10_ concentration [3–5]. The relevance of CoQ_10_ is not limited to its role in the electron transport chain but also to its antioxidant capacity. In fact, the reduced form CoQ_10_H_2_ is an efficient chain breaking antioxidant able to inhibit lipid peroxidation by reacting with carbon- and oxygen-centred radicals [6–8] and by recycling Vitamin E from its one electron oxidation product, the tocopheroxyl radical [9,10]. The interaction of CoQ_10_ and CoQ_10_H_2_ with superoxide anion (O_2_^−•^) may also be important [7,11] because O_2_^−•^ is the proximal radical produced during oxidative stress within mitochondria [12]. Moreover, CoQ_10_H_2_ can react also with Reactive Nitrogen Species (RNS) which are both endogenous products and dangerous pollutants. CoQ_10_H_2_/nitrogen monoxide (nitric oxide, ^•^NO) reactivity was kinetically studied [13] as well as CoQ_10_H_2_ oxidation by peroxynitrite (^−^OONO) [14]. In both cases, nitrosative damage was prevented by the one electron oxidation of CoQ_10_H^–^ anion to CoQ_10_H^•^ ubisemiquinone radical by ^•^NO or ^–^OONO: these reactions are not simple and are part of a complex mechanism with several implications for mitochondrial function and integrity.

Recently, we studied the reactivity of CoQ_10_ and of other model compounds toward nitrogen monoxide, in the presence and in the absence of oxygen, and observed the formation of nitration products [15]. In particular, they were dinitro compounds and nitroalcohols all deriving from the addition of ^•^NO_2_ to the double bonds of the isoprenic chain. Even if addition of ^•^NO_2_ to a double bond is a well-described process [16], we demonstrated for the first time that such reaction effectively occurs also between CoQ_10_ and nitrogen dioxide (^•^NO_2_). Noteworthy was the formation of a di-*tert*-butyl nitroxide, whose three lines signal [17] was clearly visible when the reaction mixtures were analysed by Electron Paramagnetic Resonance (EPR) spectroscopy. In fact, it was the first time that a nitroxide radical, already observed in the reaction between ^•^NO_2_ with alkenes [18], was obtained starting from CoQ_10_.

These findings prompted us to deepen the understanding of nitrogen dioxide (or of nitrogen monoxide/nitrogen dioxide mixture) reactivity toward CoQ_10_, naturally contained or incorporated, in biological systems. In this paper, we describe the reactions carried out on liposomes with incorporated CoQ_10_, and on mitochondria and plasma, in which CoQ_10_ is naturally present. Data concerning the rate constant of nitroxide formation in the reaction of CoQ_10_ with ^•^NO_2_ are also reported.

## Materials and methods

### Materials

Coenzyme CoQ_10_, lead (IV) nitrate Pb(NO_3_)_2_ and all the other reagents and solvents were purchased from Sigma-Aldrich and used without further purification. 1,2-Dioleoyl-*sn*-glycero-3-phosphocholine (DOPC) was purchased from Avanti Polar Lipids as chloroform solution.

### Preparation of liposomes

Liposomes were prepared by the ‘thin film hydration’ method. Appropriate amounts of chloroform solutions of DOPC (200 mM) and CoQ_10_ (2 mM) were mixed in a 200:1 molar ratio. The solvent was slowly evaporated with a stream of nitrogen and the thin film obtained was dried for at least 2 h under reduced pressure. This dried film was then resuspended by vortex agitation in the required amount of 5 mM PB (pH 7.4) to a 50 mM DOPC and 0.25 mM CoQ_10_ final concentration and incubated overnight to swell and stabilize. The resulting liposomes were sonicated by a probe sonicator (Sonic Vibracell) for 10 min at 50% amplitude.

### Preparation of liver bovine mitochondria

Bovine liver mitochondria were isolated from 100 to 150 g fresh liver of adult bovine supplied by a local slaughterhouse. Mitochondria were purified from liver tissue, clean washed and homogenized in homogenization buffer pH 7.5 (1:10 w:v), containing 75 mM sucrose, 225 mM mannitol, 1 mM EDTA, 5 mM Hepes and 0.5 mg/mL bovine serum albumin fatty acid free. The obtained homogenate was centrifuged for 10 min at 600 *g* at 4°C. Subsequently, the supernatant was centrifuged for 20 min at 1200 *g* at 4°C. The mitochondrial pellet was washed two times at 2800 *g* for 10 min at 4°C and purified mitochondria were carefully resuspended in 1 mL ice-cold homogenization buffer [19]. The mitochondrial protein content was determined by the Bradford protein assay [20]. Purity of isolated mitochondria was checked by measuring Glo I activity, a cytosolic marker, in the mitochondrial suspension. Enzymatic activity was determined spectrophotometrically at 240 nm using 1.0 mM GSH/methylglyoxal hemithioacetal as substrate in 100 mM sodium phosphate buffer, pH 6.6. The hemithioacetal is generated *in situ* by pre-incubation of 2 mM methylglyoxal with 2 mM GSH in 100 mM sodium phosphate buffer pH 6.6 at 37°C and this step is essential to avoid conditions where the formation of the hemithioacetal is rate limiting.

### Preparation of plasma

Human blood was withdrawn from a volunteer (one of the authors), collected into heparinized tube and kept in the refrigerator overnight. Plasma was obtained by spontaneous decantation and used without any treatment. In the case of orally administrated CoQ_10_, 3 × 100 mg CoQ_10_ pills were administered every 5 h starting at 8.00 am by the volunteer and the withdrawing was performed at 10.00 pm. Typical CoQ_10_ plasma levels were 0.8 μg/mL before and 3 μg/mL after CoQ10 administration as verified by HPLC using the method described in [21].

### General procedures

#### Reactions with ^•^NO_2_

Nitrogen dioxide was obtained from the thermal decomposition of Pb(NO_3_)_2_ and bubbled into the sample (liposomes, mitochondria or plasma) for 2 min under stirring. The reaction mixture was immediately transferred into a Pasteur pipette and inserted into the EPR cavity for the measurements.

#### Reaction with ^•^NO/^•^NO_2_.

The ^•^NO/^•^NO_2_ mixture was obtained from nitrous acid decomposition in a three-way distillation receiver equipped with three receiver flasks. Sodium nitrite (0.5 g) was poured in one of the flasks, acetic acid (2 mL) in the second and the biological sample (1 mL) in the third one. The apparatus was closed in the presence of air and acetic acid was poured into the NaNO_2_ compartment avoiding the contact with the sample. From this mixture nitrous acid was formed, but it decomposed into nitrogen monoxide which reacted with oxygen giving a ^•^NO/^•^NO_2_ mixture. The sample (liposomes or mitochondria) was exposed to this gaseous atmosphere for 30 min before EPR measurements.

### EPR measurements

EPR spectra were recorded on a Bruker EMX EPR spectrometer equipped with an XL microwave frequency counter, Model 3120 for the determination of *g*-factor and on a Varian E4 spectrometer. The spectra were recorded with the following instrumental settings: modulation frequency 100 kHz, modulation amplitude 0.4 G, sweep width 80 G, microwave power 5 mW, time constant 1.28 s, receiver gain 5 × 10^3^, number of scans 50.

*Liposomes*: 300 μL of ^•^NO_2_ or ^•^NO/^•^NO_2_ treated liposomes suspension were transferred into a Pasteur pipette and the spectrum recorded. To the same sample, 200 μL of CHCl_3_ were added and the spectrum was recorded again. 300 μL of untreated liposomes were used as a control.

*Mitochondria*: 300 μL of ^•^NO_2_ or ^•^NO/^•^NO_2_ treated mitochondria suspension were transferred into a Pasteur pipette and the spectrum recorded. 500 μL of these treated mitochondria suspensions were treated with a Triton X solution and extracted with CHCl_3_ (2 × 4 mL). The collected chloroform solution was reduced to 2 mL volume under reduced pressure and then submitted to EPR spectroscopy.

*Plasma*: nitrogen dioxide was bubbled into 500 μL of plasma until complete solidification of the sample. The triturated solid was extracted with CHCl_3_ (2 × 4 mL), the volume reduced to 2 mL under reduced pressure and then submitted to EPR spectroscopy.

### Kinetic measurements

EPR spectra were recorded on a Bruker Elexsys at room temperature. ^•^NO_2_ solution was obtained by bubbling ^•^NO_2_ (thermal decomposition of Pb(NO_3_)_2_) in cyclohexane for 30 s; its concentration was determined by weighting the solution before and after bubbling. ^•^NO_2_ 100 mM solution (100 μL) was added to a 6.5 mM solution of CoQ_10_ (200 μL) in an NMR tube (final concentration [CoQ10] = 4.3 mM, [^•^NO_2_] = 36 mM), vortexed and introduced in the EPR cavity 65 s after mixing; 500 scans (1 s per scan) were recorded with a delay of 2 s between each scan. Nitroxide concentration was determined from a calibration curve obtained from TEMPO solutions (10^−6^–10^−4 ^M).

## Results

**Figure 1.**
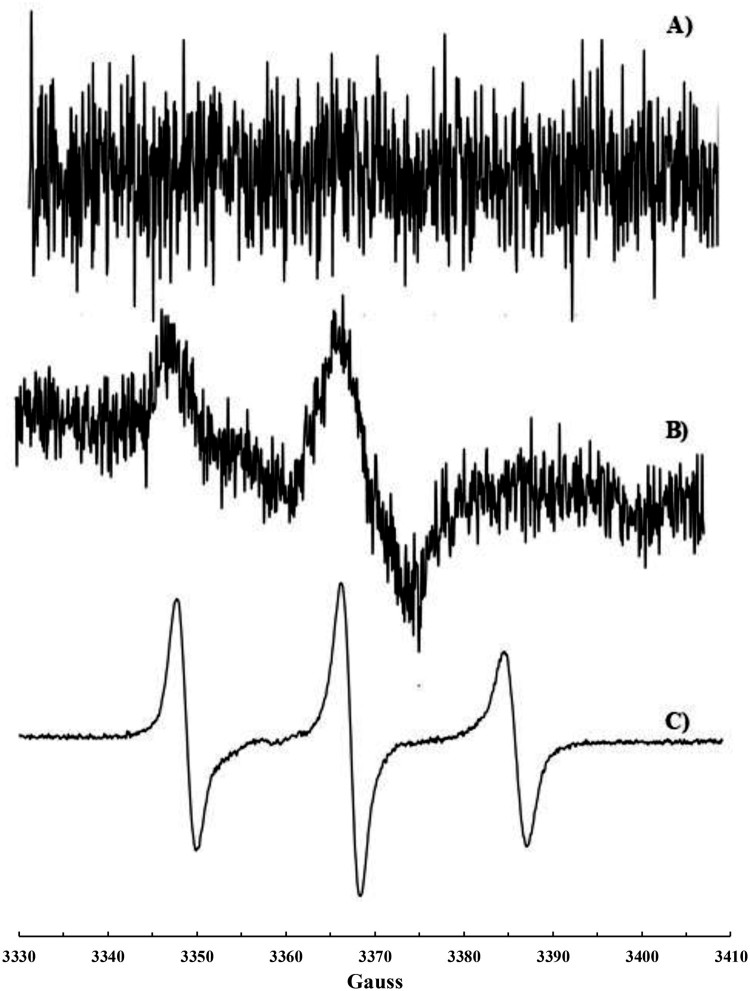
EPR signals recorded on liposomes suspension containing CoQ_10_: (a) untreated; (b) treated with ^•^NO_2_ or ^•^NO/^•^NO_2_; (c) treated with ^•^NO_2_ or ^•^NO/^•^NO_2_ and extracted with CHCl_3_.

**Figure 2.**
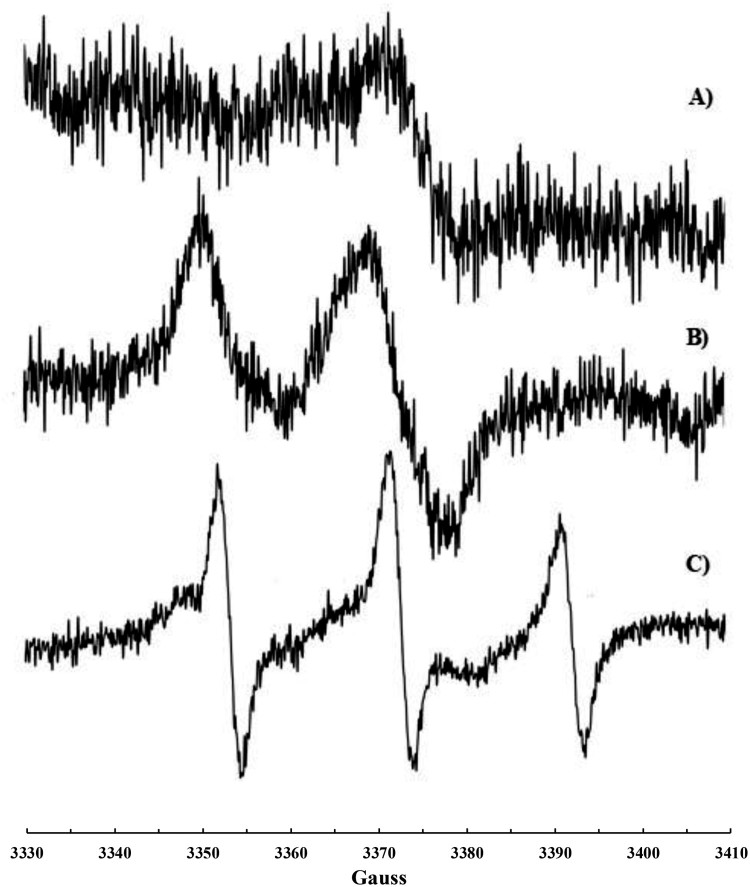
EPR signals recorded on mitochondria suspension: (a) untreated; (b) treated with ^•^NO_2_ or ^•^NO/^•^NO_2_; (c) treated with ^•^NO_2_ or ^•^NO/^•^NO_2_, lysed with Triton X-100 and extracted with CHCl_3_.

**Figure 3.**
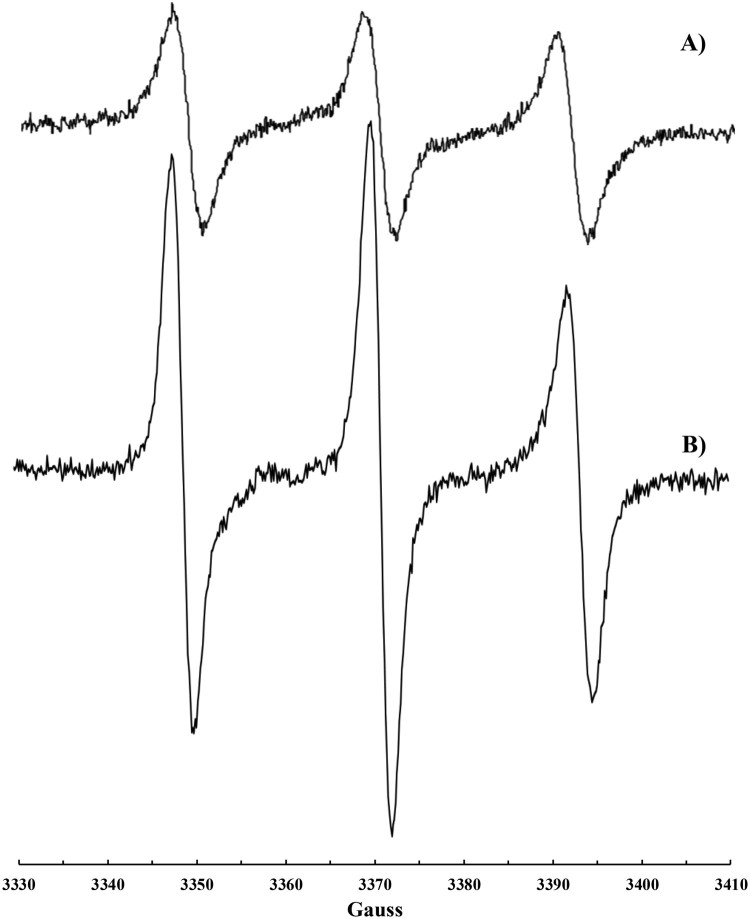
EPR signal recorded on human plasma treated with ^•^NO/^•^NO_2_ after addition of CHCl_3_: (a) plasma sample and (b) plasma sample collected after CoQ_10_ administration.

Before considering the biological systems object of this study, the reaction between a chloroform solution of CoQ_10_ and ^•^NO_2_ or a mixture of ^•^NO/^•^NO_2_ was repeated and followed by EPR spectroscopy. Nitrogen dioxide (^•^NO_2_) was produced by thermal decomposition of lead (IV) nitrate [Pb(NO_3_)_2_], whereas ^•^NO/^•^NO_2_ mixture was obtained by spontaneous decomposition of nitrous acid generated form sodium nitrite and acetic acid in the presence of oxygen (see experimental). In fact, ^•^NO_2_ is rapidly formed (*k* = 1.1 × 10^6 ^M^−1 ^s^−1^) [22] from the reaction between ^•^NO and oxygen. The typical three lines EPR signal already reported by us [15] characterized by *a*_N_ = 15.4 Gauss, g-factor 2.0062_(3)_ was recorded, and it was identical to those shown in Figures 1(c), 2(c) and 3(b).

### Reaction of ^•^NO_2_ or ^•^NO/^•^NO_2_ mixture with CoQ_10_-containing liposomes

**Scheme 2.**
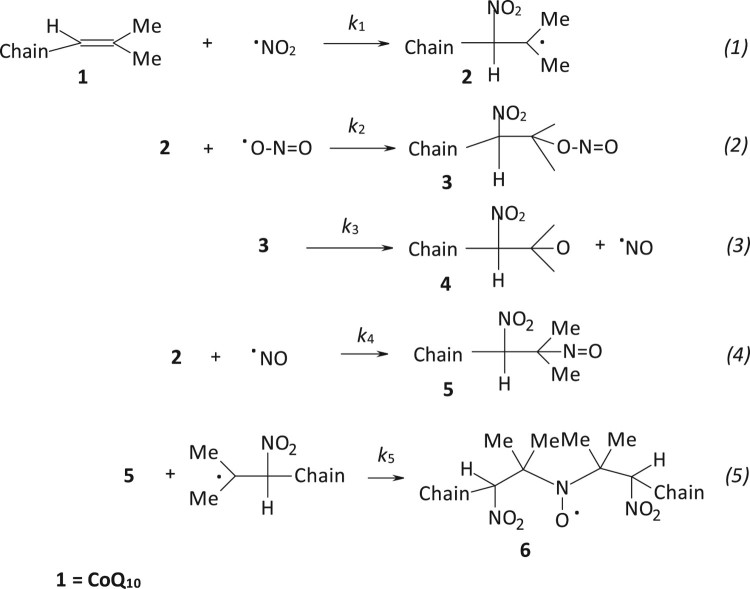
Sequence of reactions responsible for nitroxide **6** formation.

1,2-Dioleoyl-*sn*-glycero-3-phosphocholine (DOPC) liposomes were prepared and supplemented with CoQ_10_. No EPR signal was recorded on these liposomes before exposure to ^•^NO_2_ or ^•^NO/^•^NO_2_ (Figure 1a). After exposure to ^•^NO_2_ or ^•^NO/^•^˙NO_2_ mixture, a very weak signal was recorded and shown in Figure 1(b): it is the typical signal of a nitroxide immobilized in a high viscosity region such as the lipid bilayer of DOPC liposomes. Chloroform was then added to dissolve the lipid and extract the nitroxide formed. A very well-resolved three lines signal (*a*_N_ = 15.58 G, *g* = 2.0062_(3)_) was recorded (Figure 1c) and assigned to a di-*tert*-alkyl nitroxide formed upon addition of ^•^NO_2_ to the carbon–carbon double bond of the isoprenic chain according to the mechanism already described [15] and reported in Scheme 2.

### Reaction of ^•^NO_2_ or ^•^NO/^•^NO_2_ mixture with mitochondria

The same kind of experiment was carried out on isolated bovine liver mitochondria. Also in this case, the typical signal for an immobilized nitroxide (Figure 2b) was detected when mitochondria suspensions were introduced into the EPR cavity after treatment with ^•^NO_2_ or ^•^NO/^•^NO_2_ mixture. The addition of Triton X-100 solution to the suspension resulted in mitochondria lysis and extraction of the di*-tert*-alkyl nitroxide with chloroform was then possible. A well-resolved three lines EPR signal (*a*_N__ _= 15.65 G, *g* = 6.0062_(3)_) was recorded (Figure 2c), even if a second radical species may be glimpsed in this spectrum, likely another nitroxide of the same type formed upon reaction of ^•^NO_2_ with a different double bond of the isoprenic chain. In fact, the overlapping of more signals was observed when CoQ_10_ solutions were reacted with increasing amounts of nitrogen dioxide [15]. EPR measurements were carried out also before treatment with ^•^NO_2_. A very weak signal was recorded (Figure 2a) likely due to a naturally occurring nitroxide formed upon reaction between CoQ_10_ (present in mitochondria) and endogenously produced ^•^NO_2_.

### Reaction of ^•^NO_2_ or ^•^NO/^•^NO_2_ mixture with human plasma

Nitroxide formation (*a*_N_ = 15.41 G, *g*-factor 2.0062_(5)_, Figure 3a) was observed also upon exposure of plasma to ^•^NO_2_ or to a ^•^NO/^•^NO_2_ mixture. In this case, the addition of CHCl_3_ was necessary to dissolve the solid formed immediately after the addition of ^•^NO_2_ to plasma. A more intense signal (Figure 3b) was recorded on plasma samples if some CoQ_10_ was orally administrated to the volunteer in the days before the blood withdrawing.

### Kinetic measurements

**Figure 4.**
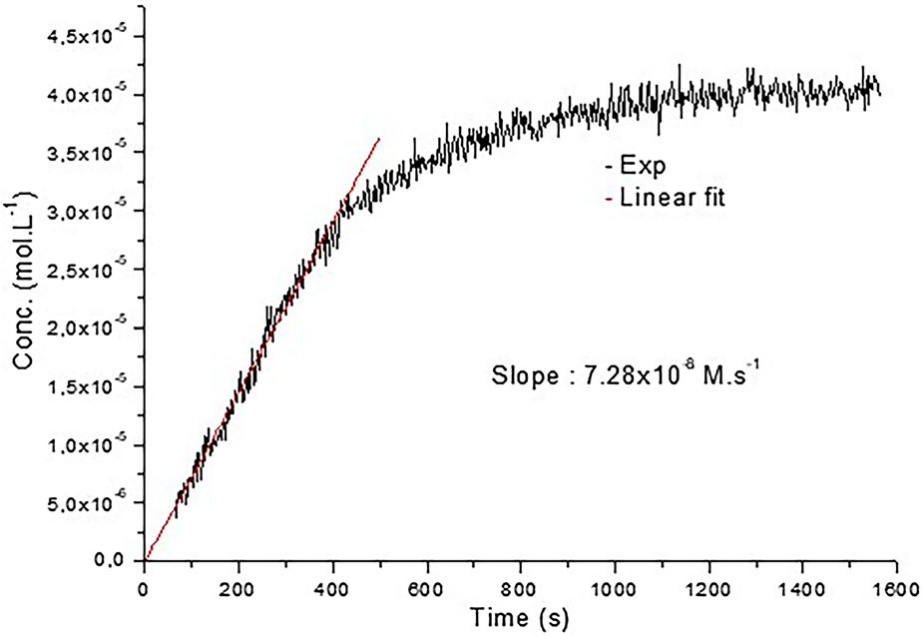
Kinetic of the reaction CoQ_10_ + ^•^NO_2_. Linear fit was done for the 400 first seconds leading to a *k* = 7.28 × 10^−8 ^M s^−1^.

An EPR signal was observed upon mixing of cyclohexane solutions of CoQ_10_ and ^•^NO_2_ (final concentration [CoQ_10_] = 4.3 mM, [^•^NO_2_] = 36 mM, at *t* = 0) and monitored over time (Figure 4). The evolution of nitroxide **6** concentration showed a linear increase within the first 400 s with a slope of 7.28 × 10^−8 ^M s^−1^ resulting from a limiting step in the cascade reactions reported in Scheme 2.

## Discussion

The obtained results confirm the previous findings that ^•^NO_2_ may react with the double bonds of the CoQ_10_ isoprenic chain [15], though a very complex process with formation of nitroxide **6** together with other nitration products. Moreover, they demonstrate that such reactions may take place also in biological systems such as liposomes (upon incorporation of CoQ_10_) or in mitochondria and blood where CoQ_10_ is naturally occurring. The various steps necessary to justify the formation of the nitroxide are reported in Scheme 2 and some considerations can be drawn concerning their kinetics. The addition of ^•^NO_2_ to the terminal double bond of the isoprenic chain (Equation 1, Scheme 2), which is the most accessible and less sterically hindered, has a calculated (DFT) activation energy of 7.9 kcal [15], and the rate constant for this reaction should be in the range of 10^4^–10^6 ^M^−1 ^s^−1^ in agreement with the value of 10^6 ^M^−1 ^s^−1^ reported for the addition of ^•^NO_2_ to double bonds of arachidonic acid [23]. Reactions occurring in steps 2, 4 and 5 are very fast, being free radical couplings (steps 2 and 4) and radical addition to a nitroso compound (step 5) with a rate constant of about 10^6 ^M^−1 ^s^−1^ [24]. It follows that the slowest reaction in the proposed process should be the decomposition of the alkyl nitrite compound **3** in step 3 with formation of ^•^NO. This hypothesis is confirmed by the slow decomposition rate constants reported for some alkyl nitrites (1 mM in 0.1 M phosphate buffer at 37°C) in the range 2.7 × 10^−8^–1 × 10^−7 ^M s^−1^ [25]. Since step 3 is the limiting reaction, nitroso compound **5**, formed upon addition of ^•^NO to radical **2**, should always be at a low concentration compared to **2** and hence nitroxide **6** should form with a rate (7.28 × 10^−8 ^M s^−1^ as determined in the kinetic experiment) close to alkyl nitrites decomposition rate [25]. Although the formation of **6** is slow and derives from a complex process with competitive side reactions such as that of ^•^NO with O_2_ or the self-coupling of radical **2** (not considered in that study), we believe that the formation of this nitroxide is an indirect proof of the possible reaction of ^•^NO_2_ with the isoprenic chain of CoQ_10_. Moreover, the fact that an EPR signal, even if very weak, likely due to a nitroxide like **6** was recorded on untreated mitochondria and that CoQ_10_ is present in various organs [26] could make this reaction relevant for biological media.
